# Metagenomic Approaches for Public Health Surveillance of Foodborne Infections: Opportunities and Challenges

**DOI:** 10.1089/fpd.2019.2636

**Published:** 2019-07-09

**Authors:** Heather A. Carleton, John Besser, Amanda J. Williams-Newkirk, Andrew Huang, Eija Trees, Peter Gerner-Smidt

**Affiliations:** Enteric Diseases Laboratory Branch, Division of Foodborne, Waterborne and Environmental Diseases, Centers for Disease Control and Prevention, Atlanta, Georgia.

**Keywords:** foodborne disease surveillance, metagenomics, whole-genome sequencing

## Abstract

Foodborne disease surveillance in the United States is at a critical point. Clinical and diagnostic laboratories are using culture-independent diagnostic tests (CIDTs) to identify the pathogen causing foodborne illness from patient specimens. CIDTs are molecular tests that allow doctors to rapidly identify the bacteria causing illness within hours. CIDTs, unlike previous gold standard methods such as bacterial culture, do not produce an isolate that can be subtyped as part of the national molecular subtyping network for foodborne disease surveillance, PulseNet. Without subtype information, cases can no longer be linked using molecular data to identify potentially related cases that are part of an outbreak. In this review, we discuss the public health needs for a molecular subtyping approach directly from patient specimen and highlight different approaches, including amplicon and shotgun metagenomic sequencing.

## Introduction

Within the last few years, a revolution has taken place in clinical testing. Clinical diagnostic laboratories are increasingly moving away from traditional culture-based methods to culture-independent diagnostic tests (CIDTs) which are molecular tests that identify pathogens directly in the specimen without producing an isolate (Atkinson *et al.*, 2013; Iwamoto *et al.*, 2015).

Among the first CIDTs were enzyme immunoassays (EIAs) detecting single or multiple pathogens. Among foodborne enteric bacterial pathogens, the most notable EIA was developed for the detection of Shiga toxins in *Escherichia coli*. The widespread use of this assay revealed that a substantial portion of Shiga toxin–producing *E. coli* (STEC) infections were caused by non-O157 serotypes not easily recognized in culture. However, CIDTs for enteric pathogens gained popularity during the past decade when polymerase chain reaction (PCR)-based “syndromic” panels were commercialized, permitting detection of up to 22 bacterial, viral, and parasitic pathogens in a stool sample in a matter of hours.

These tests are highly accurate and are performed on minimally processed patient specimens. Thus, these tests can provide clinically actionable information more rapidly than gold standard culture-based identification techniques. However, unlike gold standard methods, the CIDTs do not yield a pure culture of the pathogen. For reportable pathogens such as *Salmonella enterica*, *Listeria monocytogenes*, and STEC, cultured isolates are forwarded to local and state public health laboratories to perform subtyping for public health surveillance. Although these subtyping results rarely contribute to direct patient care, subtype information is critical for public health surveillance and enteric disease outbreak detection.

Subtypes of foodborne pathogens are compared at the local, state, and national levels through PulseNet, the national foodborne molecular surveillance system. Outbreaks caused by nationally or regionally distributed products or from local food handling problems can be identified by using subtype information to group together case patients likely to have a common exposure, such as a common food source. Annually, PulseNet processes subtype data from about 70,000 isolates of clinical foodborne pathogens from public health partners, and include data streams from human and animal diagnostics as well as regulatory testing of food and food production environments.

PulseNet's routine, systematic use of subtype data from isolates at state and local public health laboratories has a tremendous public health impact, preventing an estimated 270,000 illnesses annually and saving the U.S. economy more than 500 million U.S. dollars (Scharff *et al.*, 2016). Since current surveillance depends on the availability of isolates, the increasing use of CIDTs by clinical laboratories is compromising molecular surveillance and outbreak detection of foodborne illness. Efforts are underway to encourage reflex culture of CIDT-positive specimens, but this process is slow, expensive, and unlikely to be sustainable in the long run. To adapt to the new clinical laboratory workflow with CIDTs, new diagnostic and subtyping techniques that can be performed directly on clinical specimens without the need for culture must be developed.

These new specimen-based subtyping approaches must meet certain requirements to be effective in a public health surveillance system. Most importantly, specimen-based approaches must be rapid, affordable, and highly accurate. Ideally, their cost should not exceed the cost of the current subtyping approach, whole-genome sequencing (WGS), which costs 95–300 U.S. dollars in materials per isolate, depending on the workflow in the laboratory. In addition, the laboratory and analytical workflow needs to be completed in 3–4 working days and be compatible with a standard 8-h workday and minimal hands-on time.

For the laboratory workflow, all sequencing should use existing next-generation sequencers in public health laboratories; new equipment should entail minimal equipment costs and service contract expenses. Analytical tools must be user-friendly and push-button because a majority of laboratories have limited information technology (IT) and bioinformatic support and limited, if any, access to high-performance computing. Critically, the final subtyping output must be able to distinguish outbreak from background sporadic isolates at both a local and national level and link specimens from epidemiologically related cases.

Ideally, the output should also be compatible with isolate-based analysis workflows used to identify and/or confirm potential outbreaks and outbreak sources, and it should ensure historical compatibility with isolate-based WGS data. Given these requirements and limitations, the ideal solution for public health surveillance for foodborne infections would be a user-friendly laboratory procedure generating sequence data directly from the primary specimen that could be analyzed using push-button bioinformatic tools that are easily deployable in a public health laboratory.

**FIG. 1.**
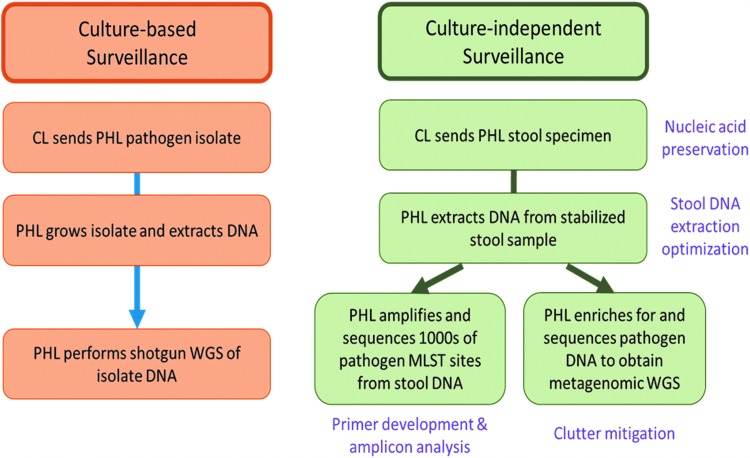
Comparison of isolate based on culture-independent public health surveillance workflows. CL, clinical laboratory; PHL, public health laboratory.

Unfortunately, there is no single existing specimen-based subtyping approach that can meet all these requirements (Fig. 1). Here we describe the different approaches and highlight some of the exploratory work the Enteric Diseases Laboratory Branch at the Centers for Disease Control and Prevention is performing on these two approaches.

## Amplicon Sequencing

A classic example of the amplicon sequencing approach is the 16s rDNA sequencing, typically used to assess microbial diversity in a specimen. However, this approach only provides resolution at the genus or species level, limiting its utility for diagnostic and public health purposes (Poretsky *et al.*, 2014). An alternate targeted amplicon approach, highly multiplexed amplicon sequencing (HMAS) panels, potentially provides greater resolution and uses existing technology, making deployment to public health laboratories possible in a short time frame. These panels use hundreds to thousands of taxon-specific primer pairs to selectively amplify informative regions from the pathogen genome. The resulting amplicons are then sequenced. There are several laboratory methods that can affordably perform the multiplexed amplification on a single specimen using either chemical or mechanical means to facilitate the simultaneous reactions. To implement this laboratory workflow, it may require the purchase of additional equipment and reagents.

The design of HMAS panels requires a well-curated pathogen-specific database to identify the genomic regions needed to distinguish an outbreak from sporadic cases. A database has been developed for *Salmonella* from isolate-based sequencing for surveillance and is being used to design a core genome multilocus sequence typing (cgMLST) scheme. The target regions for the HMAS panel must be flanked by pathogen-specific primer sites and be of the correct length to be compatible with the intended sequencing platform.

The flexibility of HMAS panels allows additional informative targets, such as antimicrobial resistance (AMR), virulence, and serotyping genes, to be added to the subtyping amplicon panel. Existing HMAS panel laboratory methods are also amenable to sample multiplexing, allowing simultaneous amplification of several specimens and loading of the products onto a sequencing platform with minimal processing. Analysis will require the development of new or repurposed existing open-source bioinformatic pipelines. Our *in silico* studies of amplicons generated from isolate genome assemblies have shown that it is possible to subtype *Salmonella* at a resolution similar to that of the PulseNet cgMLST scheme using ∼1500 loci (unpublished data).

While amplicon sequencing is a mature technology, there are several obstacles to implementing HMAS panels at scale. One challenge is the need for large numbers of primers specific to the pathogen of interest, for example, *Salmonella*, in extremely complex and bacteria-rich clinical specimen backgrounds such as stool. In addition, some enteric pathogens are closely related to the commensal gut microflora, making it difficult to identify enough pathogen-specific targets for the high-resolution subtyping required for foodborne outbreak surveillance. For example, commensal *E. coli* frequently co-occurs in stool specimens with STEC, but virulence genes are the only markers known to distinguish STEC from other strains of *E. coli*. Unfortunately, virulence genes alone do not contain enough information to identify outbreak-related strains.

**FIG. 2.**
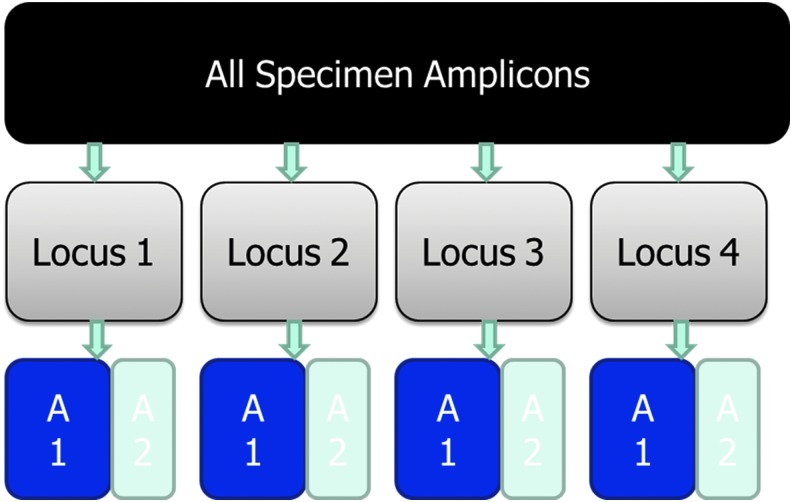
Disambiguation of amplicon targets in highly multiplexed amplicon sequencing. Using single-copy pathogen and species target for Shiga toxin–producing *Escherichia coli* and *E. coli* to obtain genome copy number, algorithms can be used to classify gene locus variants as belonging to pathogen (A1) or commensal (A2).

One potential approach is to computationally distinguish reads that belong to the pathogen versus commensal bacteria by amplifying only *E. coli*-specific single-copy core genes. The different alleles for each gene will then appear in the sequencing data in the same proportion as the strain of origin in the *E. coli* population of the patient sample. These proportions can be used to group alleles from different genes by their strain of origin. This concept is illustrated in Figure 2 and one potential implementation is described in Mustonen *et al.* (2018). This approach may also distinguish whether reads generated from mutational AMR determinants belong to the pathogen or background bacteria since studies have shown that health adults carry AMR genes in their gut microflora (Fitzpatrick and Walsh, 2016).

A second obstacle to the use of HMAS panels in surveillance subtyping is the challenge of calling alleles when multiple amplicons are required to span a single informative region. The problem becomes even more complex when the metagenomic sample contains multiple alleles for the same locus. Existing tools for calling genes from shotgun metagenomic data such as ROCker (Orellana *et al.*, 2016) and Kraken (Wood and Salzberg, 2014) may be adapted to resolve this problem. However, despite the challenges in the laboratory workflow and bioinformatic analysis, HMAS fits our public health subtyping requirements of low cost per specimen, high data resolution, and rapid laboratory and bioinformatic turnaround times.

There are additional targeted approaches that either enrich target pathogen DNA in the sequencing library or deplete nontarget DNA. Depletion of nontarget DNA before sequencing could include both human and other eukaryotic and prokaryotic DNA in the stool. Techniques for the removal of human DNA rely primarily on differential lysis of cell types or selective binding of CpG-methylated DNA (Stevens and Jaykus, 2004; Bachmann *et al.*, 2018; Marotz *et al.*, 2018; Velasquez-Mejia *et al.*, 2018). Once again, the presence of closely related nontarget commensal bacteria in the sample makes it challenging to target only pathogenic strains.

One targeted depletion approach is called DASH (Depletion of Abundant Sequences by Hybridization) (Gu *et al.*, 2016). DASH relies on a Cas9 approach to guide RNAs to target and cleave known high-abundance background DNA, such as human host material. The DASH approach was shown to successfully enrich pathogen sequence reads in a cerebrospinal fluid (CSF) metagenomics through depletion of human background in the sample, although CSF is a less complex sample than stool and DASH has not been tested in stool metagenomic samples.

**FIG. 3.**
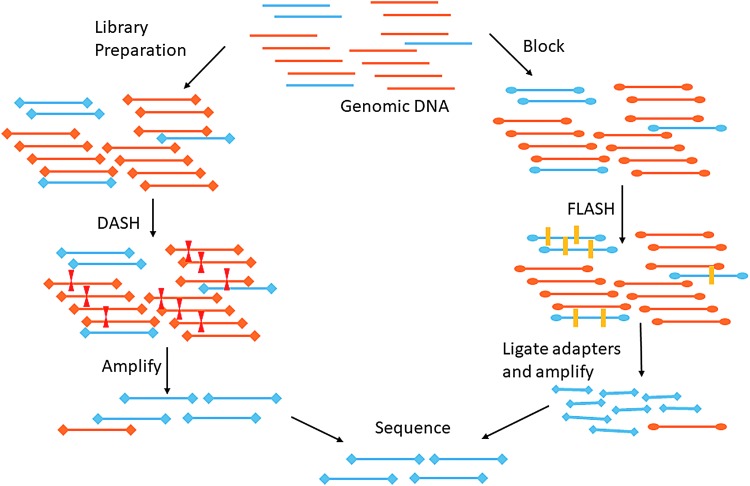
CRSIPR-Cas9 DASH and FLASH approaches to targeted sequencing. DASH targets background abundant sequences (in orange) for cleavage by Cas9. The targeted library no longer has adaptors on both ends of the sequence and is not subsequently amplified and sequenced. FLASH-Cas9 approach cleaves targeted low-abundant DNA that is subsequently available for adaptor ligation, amplification, and sequencing. DASH, Depletion of Abundant Sequences by Hybridization; FLASH, Finding Low Abundance Sequences by Hybridization.

Another Cas9-based approach, FLASH (Finding Low Abundance Sequences by Hybridization), targets DNA to enrich for sequencing (Quan *et al.*, 2018). This requires input of enrichment targets, which can include AMR genes or other known subtyping targets. The initial study demonstrated enrichment of AMR targets on respiratory metagenomic samples. Both Cas9 approaches have low hands-on time in the laboratory and a low cost per sample after the initial guide RNA generation costs. The methods are briefly outlined in Figure 3.

One challenge for FLASH is that the high specificity of the approach may require individual guides to be generated for each targeted sequence variant to enrich for all of the possible subtypes. This contrasts with PCR primers, which have some template mismatch tolerance. Other target enrichment approaches rely on the generation of a biotinylated bait library to target specific pathogen library DNA to enrich those libraries for subsequent sequencing workflows (Briese *et al.*, 2015). There has been some initial promising results from a pilot study targeting STEC using a custom-designed bait capture approach from stool specimens (Singh *et al.*, 2019). There are also a few companies (Roche, Agilent, Arbor, and IDT) that offer probe generation as a commercial service and provide kits for the subsequent laboratory workflow (Depledge *et al.*, 2011, 2014). Although these commercial approaches work well for enrichment of targeted and similar DNA sequences, particularly for viral and cancer-specific targets, the workflow's cost and labor intensiveness are challenging for public health.

## Shotgun Metagenomics

The other specimen-based subtyping approach, shotgun metagenomics, is used to sequence all DNA in the specimen in an unbiased manner, from which pathogen signals can be isolated and characterized. Although this approach is more costly and with limited throughput, advances in information and sequencing technology may bring the cost down to meet the requirements of a public health molecular surveillance system (Scholz *et al.*, 2012). One advantage to this approach is that it is agnostic to the pathogen, so you do not need *a priori* knowledge about the target microbe, which is helpful if the specimen contains multiple or novel pathogens. Even if the agent is not detected by the initial CIDT panel, unculturable, or unknown, shotgun metagenomics can detect and identify such pathogens.

A few proof-of-principle studies have been published proving the utility of this approach (Loman *et al.*, 2013; Huang *et al.*, 2016). A significant challenge with this approach is the large variation in the pathogen load between patient samples, which means that classical shotgun metagenomic sequencing alone may only generate a few sequence reads from the target pathogen when pathogen loads are low. Hence, there may not be enough data to produce the high-resolution subtype needed to distinguish and cluster the cases that were caused by the same outbreak pathogen source.

In addition, there are few bioinformatic pipelines that produce the resolution needed for strain-level characterization from metagenomic reads. Most shotgun metagenomic analysis pipelines provide resolution to the species level, but not to the subtype level (Wood and Salzberg, 2014). Finally, there are also challenges in distinguishing reads associated with the pathogens versus the commensal microflora.

Emerging approaches for binning metagenomic reads and contigs using reference-based and reference-free approaches are making it possible to sort sequences into species-based bins for further analysis (Sangwan *et al.*, 2016). Reference-based binning approaches, such as MIDAS, may be useful in cases where a common pathogen is already suspected via CIDTs or may be useful in filtering known organisms from metagenomic data sets (Gaunt *et al.*, 2006). For metagenomic data sets that may contain unknown pathogens in common, reference-free approaches, such as MetaBAT and MaxBin, may be able to bin sequences by species using nucleotide composition and read abundance, which can also aid in more complete genome recovery (Kang *et al.*, 2015; Wu *et al.*, 2016).

Moreover, both binning approaches are able to take advantage of whole sets of samples as might be found in presumptive clusters during an outbreak investigation. By focusing on genomic material in common among samples, the output from these programs can be used to generate a phylogenetic tree showing the relatedness of samples using only the common sequence bins, which may correspond to shared pathogens (Nayfach *et al.*, 2016; Costea *et al.*, 2017).

There are some laboratory-based approaches to link together sequences generated from the same organism found in a specimen. One approach includes crosslinking DNA from the same cell and then using the markers left by crosslinking to bioinformatically link reads generated by the same cell. Phase Genomics and others have commercialized this approach, and it has been successful in human gut metagenomic samples (Press *et al.*, 2017). Although promising, the additional laboratory processing needed and the computationally intensive proprietary bioinformatic methods required to deconvolute the data make it currently impractical in a public health workflow.

Another promising laboratory approach is low-cost long-read metagenomic shotgun sequencing. For example, Oxford Nanopore's technology can generate 15–100 kB reads, which are long enough to physically link pathogen marker genes, such as the Shiga toxin phage region of STEC, with other core genes useful in subtyping from the surrounding regions.

There are several proof-of-principle studies that have shown the utility of long-read sequencing from metagenomic samples, although these approaches focus on pathogen identification or identification of resistance genes, rarely strain level subtyping (Lemon *et al.*, 2017; Schmidt *et al.*, 2017; Ashikawa *et al.*, 2018). The higher base error rates in long read sequence data, although constantly improving, remain a challenge for their application in foodborne pathogen surveillance because of the comparatively small number of nucleotide changes defining the typical outbreak cluster. There have been some initial studies recently that have demonstrated that platforms such as Oxford Nanopore can generate enough accurate long reads to properly place a sample in an outbreak clade, although more studies, particularly with STEC, are needed (Hyeon *et al.*, 2018).

Although real-time shotgun metagenomics for public health surveillance may not currently be feasible, clinical laboratories are also moving to metagenomic approaches as a way to identify pathogens from clinical specimens (Gu *et al.*, 2019). There are a few commercial metagenomic tests for clinical samples, including blood and CSF (Chiu and Miller, 2019). Building workflows to incorporate this type of sample processing in public health laboratories and developing bioinformatic pipelines to pull out potential strain-level data now can position public health to be at the forefront of technological advancements in the clinical space.

## Concluding Remarks

The advantages and disadvantages of the specimen-based subtyping approaches outlined in this review are highlighted in Table 1. The ideal metagenomic solution is a timely and cost-effective bedside test that serves both clinical and public health needs. This could be a small sequencer attached to an internet-connected smartphone app that analyzes shotgun metagenomic sequences as they are generated and sends reports to both the patient's physician and to the local public health laboratory. Thus, the clinician would receive microbial information needed to guide treatment of the patient, while public health professionals would be able to detect and respond to outbreak signals at the population level.

**Table 1.** Overview of Metagenomic Approaches Deployable in a Public Health Laboratory and the Potential Strengths and Limitations of Each

	*Public health requirements*					
*Metagenomic approach*	*Affordable*	*Rapid laboratory/analysis workflow*	*Requires new laboratory equipment*	*Push-button analysis tools available*	*Provides strain-level resolution*	*Historically compatible*
Highly multiplexed amplicon sequencing panel	Yes	Yes	Maybe	In development	Yes	Yes
Off-target depletion approaches: DASH	Yes	TBD	No	No	TBD	TBD
Target enrichment approaches: FLASH	Yes	TBD	No	No	TBD	TBD
Target enrichment approaches: Bait-capture	No	No	No	No	TBD	TBD
Unbiased metagenomics: reference-based analysis	No	TBD	No	No	Yes	TBD
Unbiased metagenomics: Reference-free analysis	No	TBD	No	No	Yes	No

If certain requirements have not been determined yet, then “to be determined,” TBD, is listed.

DASH, Depletion of Abundant Sequences by Hybridization; FLASH, Finding Low Abundance Sequences by Hybridization.

Such a solution has the potential to reduce turnaround time by weeks compared with current outbreak detection methods, thereby enabling a faster and more efficient public health response. If public health and food and regulatory databases contain comparable sequence data, then signals could be quickly detected for potential outbreak sources. In addition, if molecular data were linked to patient and population-based demographic and eating habit data and other food consumption information, such as shopper cards, investigators could more quickly combine the epidemiologic information and use it to confirm or disprove signals in the molecular data. Finally, as the food industry moves to higher fidelity approaches to track food from farm to fork, such as block chain, the powerful combination of these different data sources can speed up outbreak investigations and prevent more foodborne illness.

This vision cannot be achieved with the current state of sequencing, informatics, and bioinformatic technology. However, what may sound like science fiction today could become reality within the next 10–20 years.
